# Cocaine Substitutes

**Published:** 1909-11-27

**Authors:** 


### SPECIAL ARTICLE.

### COCAINE SUBSTITUTES.

There are at present a considerable number of compounds that may be used as substitutes for cocaine on account of their anæsthetic properties. Amongst these may be mentioned stovaine, novocain, tropacocaine, beta-eucaine, alypin, beta-eucaine lactate, nirvanine, holocaine hydrochloride, acoine, orthoform, and anæsthesine.

The relative value of these, as regards both their anæsthetic properties and their dangerous side-effects, have been investigated extensively in the Pharmacological Laboratory at Cambridge by Dr. Le Brocq, on behalf of the Therapeutic Committee of the British Medical Association. The conclusions he has arrived at are valuable, and of all the above cocaine substitutes he favours novocain as the most efficient and the least harmful. (*Vide* "Pharmaceutical Journal," 1909, p. 674.)

The points to which special attention has been paid are those laid down by Professor Braun as essential in estimating a local anæsthetic action. They are:—

1. A lower degree of toxicity than cocaine in proportion to its local anæsthetic power.
2. Sufficient solubility in water. The solutions should be stable—that is, they should keep without deterioration and be capable of sterilisation by boiling.
3. Absence of any sign of irritation. There should be no injury to the tissues; the local anæsthetic should be easily absorbed without causing any after-effects, such as hyperæmia, inflammation, infiltrations, or necroses.
4. Compatibility with adrenalin.
5. Rapid penetration of the mucous membrane and suitability for medullary anæsthesia.

The only exception Dr. Le Brocq would make to these postulates of Braun is that dealing with absorption. It is not obvious that easy absorption is desirable. If the drug is absorbed slowly it remains longer in contact with the nerve fibrils, and produces a more prolonged action. It has been stated that by delaying absorption, as by local vaso-constriction, we are giving the anæsthetic a longer time for action, and it is for this reason that adrenalin is injected with these substances.

If a drug can be obtained which fulfils the above conditions it can be safely said that it will supersede cocaine.

To arrive at a satisfactory conclusion it is necessary to deal with each drug separately, and to discover how closely each approaches the conditions specified by Braun.

### Solubility in Water.

Solubility in water is essential for subcutaneous and intraspinal injections. If a drug is not soluble in water to the extent of forming a 2 per cent. solution, Dr. Le Brocq has regarded it as unworthy of competing with cocaine. By examining the solubility of these drugs the list is considerably diminished, for acoine, holocaine hydrochloride, anæsthesine, orthoform (new), beta-eucaine, are all more or less insoluble, and for this reason alone unsuitable for producing local anæsthesia by injection. Beta-eucaine is not completely soluble in cold water to the extent of forming a 2 per cent. solution, but if the solution is warmed a 2 per cent. solution is readily obtained. As dusting powders these drugs may, of course, still prove useful, but he is not here concerned with that point.

Cocaine, stovaine, novocain, tropacocaine, beta-eucaine lactate, alypin, and nirvanine are freely soluble in water; their solutions are stable, and as a 2 per cent. solution they will keep for a short time without deterioration.

### Sterilisation of Solutions.

Cocaine cannot be boiled, as decomposition occurs, and the drug loses its activity and is gradually destroyed. Stovaine, novocain, beta-eucaine lactate, tropacocaine, alypin, and nirvanine can be sterilised at 115° C. if necessary, and they have the great advantage that they undergo no change, the drug being as active after as before sterilisation.

### Local Anæsthetic Properties.

The determination of the local anæsthetic action is not easy, as it is not practicable to work with accuracy on the lower animals on account of the difficulty in determining when sensation is absent or merely blunted. The method Dr. Le Brocq adopted was to take cocaine as the standard and to compare each drug with it separately.

By numerous experiments on frogs, rabbits, and human beings he arrived at the following conclusions:—

Stovaine has a more powerful anæsthetic action, weight for weight, than any of the other local anæsthetics. Alypin, beta-eucaine lactate, novocain, and tropacocaine have anæsthetic properties about equal to cocaine. Nirvanine as a local anæsthetic is inferior to cocaine.

### Toxicity.

Having determined the relative anæsthetic action, it remained to estimate the toxicity of these drugs. The method employed was to find the minimal lethal dose—the smallest dose which will kill the animal—in frogs, mice, and rabbits.

All these drugs produce death by paralysing the central nervous system in mammals and the heart in frogs. When a toxic dose of a drug is injected into a frog, movements gradually cease; the animal soon lies still in any position in which it is placed, and becomes to all external appearances dead; respiration ceases, and there is no response to any sort of stimulus. If the dose has not been large enough to paralyse the heart, it continues to beat, and as soon as the effects of the drug on the nervous system pass off the frog recovers. The explanation of the recovery is, of course, that a frog can exist for a considerable time, even for many hours, without respiration. Thus in obtaining the minimal lethal dose in frogs it is necessary to give these drugs in doses which paralyse not only the nervous system, but the heart also.

Therefore the minimal lethal dose in frogs represents the action of the drug on the heart, and in mammals its action on the central nervous system.

### Toxicity in Frogs.

The minimal lethal dose for a 20-gramme frog, using a 2-per-cent. solution, is for—

| | |
|---|---|
| Alypin* ... ... ... ... | 6 minims |
| Cocaine ... ... ... ... | 12 ,, |
| Stovaine ... ... ... ... | 15 ,, |
| Nirvanine ... ... ... ... | 17 ,, |
| Beta-eucaine lactate ... ... | 20 ,, |
| Tropacocaine ... ... ... | 20 ,, |
| Novocain† ... ... ... ... | 40 ,, |

* Most toxic. † Least toxic.

### Toxicity in Mice.

The minimal lethal dose for a 20-gramme mouse, using a 2-per-cent. solution, is for—

| | |
|---|---|
| Alypin ... ... ... ... | 4 minims |
| Cocaine ... ... ... ... | 5 ,, |
| Nirvanine ... ... ... ... | 7 ,, |
| Stovaine ... ... ... ... | 8 ,, |
| Novocain ... ... ... ... | 10 ,, |
| Tropacocaine ... ... ... | 10 ,, |
| Beta-eucaine lactate ... ... | 12 ,, |

### Toxicity in Rabbits.

The minimal lethal dose for a rabbit weighing 1,000 grammes, using a 10-per-cent. solution, is for—

| | |
|---|---|
| Alypin ... ... ... | between 18 and 23 minims |
| Cocaine ... ... ... | ,, 24 and 30 ,, |
| Stovaine ... ... ... | ,, 35 and 45 ,, |
| Tropacocaine ... ... | ,, 44 and 54 ,, |
| Novocain ... ... ... | ,, 53 and 63 ,, |
| Beta-eucaine lactate ... | ,, 65 and 75 ,, |

As the toxic action of these drugs is to paralyse the nervous system, and so the respiratory centre, the toxicity in mammals must be regarded as the correct reading, for when respiration ceases the animal dies.

If the toxicity of the cocaine be represented as 1, then—

The toxicity of alypin will be represented by the figure 1.25.

The toxicity of cocaine will be represented by the figure 1.0.

The toxicity of nirvanine will be represented by the figure 0.714.

The toxicity of stovaine will be represented by the figure 0.625.

The toxicity of tropacocaine will be represented by the figure 0.500.

The toxicity of novocain will be represented by the figure 0.490.

The toxicity of beta-eucacine lactate will be represented by the figure 0.414.

### Conclusions from the Above.

Alypin has anæsthetic powers equal to cocaine, but a higher toxicity, and so does not comply with the conditions enunciated by Braun. Nirvanine has not the anæsthetic power of cocaine, and it is only slightly less toxic. As, however, there were four drugs equal to or stronger than cocaine in anæsthetic power, and considerably less toxic than nirvanine, no further experiments with this drug were deemed necessary.

From this it will be seen that only four drugs—namely, stovaine, novocain, tropacocaine, and beta-eucaine lactate—have complied with the first two conditions. With these four drugs further experiments were performed to discover how they fulfil the other conditions laid down for the "perfect local anæsthetic."

### Irritant Action on the Tissues.

Very little is known of the action of the local anæsthetics on the tissues with which they come in contact. This effect is, however, extremely important, for it has been stated that gangrene and sloughing of the tissues have followed the use of certain of these drugs. It is well recognised, however, that they are all general protoplasmic poisons, that whilst having a special predilection for nervous structures they depress and ultimately destroy every form of living tissue.

In these, as in previous experiments, cocaine was taken as the standard. Cocaine is generally recognised as having a slight irritant action on the tissues, and occasionally, after instillation into the eye, produces considerable conjunctivitis.

For these experiments rabbits weighing about 1 kilogram were used. The abdomen was shaved and the skin washed and made aseptic, after which 10 minims of a 10-per-cent. solution of the drug was injected subcutaneously. The drug was carefully sterilised, and all antiseptic precautions were observed.

After injection the part was kept under immediate observation for several hours, and then inspected daily for one week more.

Cocaine caused slight swelling and hyperæmia soon after the injection. The part completely recovered.

Stovaine caused intense hyperæmia and dilatation of the blood-vessels, followed by sloughing of the part.

Beta-eucaine lactate caused swelling and thickening about the seat of injection, followed by sloughing.

Tropacocaine caused swelling and some thickening, followed by sloughing.

Novocain showed no swelling and no hyperæmia. The part was perfectly normal after injection, and remained so.

In the above experiments, where sloughing and necrosis occurred there were no signs of pus formation.

It is seen that a comparative investigation into the action of these drugs on the general tissue is of the utmost importance, for most of them possess a decided irritant effect; so much so that they are capable of producing necrosis and sloughing of the tissues with which they come in contact.

A 10-per-cent. solution is a stronger solution than is generally used therapeutically, yet this strength is still employed; moreover, 10 minims of a 10-per-cent. solution roughly represents 1 grain, a quantity which is far exceeded when weaker solutions are injected. And even if it be objected that it is not fair to infer from a 10-per-cent. solution what will occur in a strength of 1 per cent. or 2 per cent., yet the present experiments still show the relative irritant action of these drugs. A drug which is only mildly irritant as a 1-per-cent. solution will presumably be more irritant as a 2-per-cent. solution, until, as the strength is increased, a stage is reached when destruction of the tissues and necrosis is obtained.

The irritant action of stovaine, beta-eucaine lactate, and tropacocaine is far greater than that of cocaine; novocain is the only drug which is superior to cocaine in this respect.

### Compatibility with Adrenalin.

All the local anæsthetics are compatible with adrenalin if the solutions are fresh and kept only for a short time. After a day or two the adrenalin decomposes unless it is kept in stoppered opaque bottles.

### Conclusions.

In determining which of these four drugs is the most suitable substitute for cocaine, it is necessary to compare them with one another.

If novocain and tropacocaine be first compared, their toxicity and anæsthetic properties are, roughly, equal; but the irritant action of tropacocaine is far greater than that of novocain; in other respects their actions are similar; therefore novocain is a more suitable drug than tropacocaine.

On comparing novocain with beta-eucaine lactate it is seen that while the anæsthetic value is roughly about equal, the toxicity of the beta-eucaine lactate is slightly less than that of novocain, but the irritant action of beta-eucaine lactate is far greater than that of novocain. It appears, then, that while beta-eucaine lactate has only a slighter degree of toxicity to recommend it in preference to novocain, its irritant action far and away overshadows any such slight advantage, and novocain is recognised as undoubtedly the better drug of the two.

Finally, it only remains to compare novocain with stovaine. The former drug is less toxic and much less irritant; indeed, its specific action on nerve fibres is so great that it has practically no destructive effect on the other tissues; stovaine is more toxic and considerably more irritant.

Dr. Le Brocq comes to the conclusion, therefore, that of the drugs that have been investigated novocain is most satisfactory for general use. Its anæsthetic action is equal to that of cocaine, and its toxicity and general destructive power on the tissues are very much less.

